# Radiological Insights into UIP Pattern: A Comparison Between IPF and Non-IPF Patients

**DOI:** 10.3390/jcm14124162

**Published:** 2025-06-12

**Authors:** Stefano Palmucci, Miriam Adorna, Angelica Rapisarda, Alessandro Libra, Sefora Fischetti, Gianluca Sambataro, Letizia Antonella Mauro, Emanuele David, Pietro Valerio Foti, Claudia Mattina, Corrado Spatola, Carlo Vancheri, Antonio Basile

**Affiliations:** 1 Department of Medical Surgical Sciences and Advanced Technologies “GF Ingrassia”, University of Catania, 95123 Catania, Italy; david.emanuele@yahoo.it (E.D.); pietrofoti@hotmail.com (P.V.F.); cor_spatola@hotmail.com (C.S.); basile.antonello73@gmail.com (A.B.); 2 Pulmonary Imaging and Advanced Radiological Techniques Unit (UOSD IPTRA), 95123 Catania, Italy; mauroletizia@tiscali.it; 3 Training in Radiology Residency, Department of Medical Surgical Sciences and Advanced Technologies “GF Ingrasia”, University of Catania, 95123 Catania, Italy; miriamadorna@gmail.com; 4 Degree Course of Medicine and Surgery, University of Catania, 95124 Catania, Italy; angelica.rapisarda.ar@gmail.com; 5 Pulmonology Unit, University Hospital “Policlinico G. Rodolico-San Marco”, 95123 Catania, Italy; alessandrolibra@outlook.it (A.L.); vancheri@unict.it (C.V.); 6 Training in Pulmonology, Department of Clinical and Experimental Medicine, University of Catania, 95123 Catania, Italy; sefora.fischetti@gmail.com; 7 Department of Medicine and Surgery, University of Enna “Kore”, 94100 Enna, Italy; dottorsambataro@gmail.com; 8 Radiology 1 Unit, University Hospital “Policlinico G. Rodolico-San Marco”, 95123 Catania, Italy; 9 University Hospital Policlinico “G.Rodolico-San Marco”, 95123 Catania, Italy; claudiamattina0@gmail.com; 10 Radiotherapy UOSD, University Hospital “Policlinico G. Rodolico-San Marco”, 95123 Catania, Italy; 11 Regional Referral Center for Rare Lung Disease, Department of Clinical and Experimental Medicine, University of Catania, 95123 Catania, Italy

**Keywords:** UIP, HRCT, AI-software

## Abstract

**Background/Objectives**: This study aims to distinguish radiological differences between primary idiopathic Usual Interstitial Pneumonia (UIP) and secondary UIP patterns **Methods**: This retrospective study included patients with HRCT findings consistent with a UIP pattern. Final diagnoses were established via multidisciplinary discussion and classified as primary UIP/IPF or secondary UIP, following the 2022 ATS/ERS/JRS/ALAT guidelines. An expert thoracic radiologist (>10 years of experience), blinded to clinical data, reviewed the earliest available HRCT assessing key imaging features: honeycombing (micro-, macro- or exuberant), fibrosis distribution (symmetry, anterior-upper lobe sign, etc.), ground-glass opacities (GGO), dilatation of esophagus. Additionally, AI software AVIEW Build 1.1.46.28-win Coreline (©Coreline Soft Co., Ltd. All Rights Reserved). performed lung texture analysis, quantifying total lung volume and radiological patterns. Statistical analysis was performed to reveal results. **Results**: Among 53 cases, 31 were classified as IPF and 22 as secondary UIP cases. The expert radiologist achieved a diagnostic sensitivity of 82.9%, specificity of 889%, with a positive predictive value of 93.5%—in distinguishing between primary and secondary UIP. Primary UIP cases exhibited typical hallmark radiological features, including uniform honeycombing with cranio-caudal distribution (90.3%). Reticulations contributed significantly to the fibrotic texture, maintaining a consistent cranio-caudal gradient and axial symmetry (84.8%). Secondary UIP displayed more significant radiological heterogeneity, including patchy fibrosis with irregular GGO distribution (84.5% versus 53.33%); other findings—such as exuberant honeycombing, four corner sign and wedge-shaped fibrosis—were mainly observed in secondary pattern with respective percentages of 31.8%, 9% and 49%. **Conclusions**: Experienced thoracic radiologists, leveraging hallmark imaging features, play a critical role in improving diagnostic accuracy between primary and secondary UIP patterns.

## 1. Introduction

Usual Interstitial Pneumonia (UIP) was first described by Dr. Averill A. Liebow in the 1960s as a histopathological pattern characterized by patchy interstitial fibrosis with temporal heterogeneity, combining areas of honeycombing and fibroblast foci [1]. Initially identified through lung biopsy, its radiologic counterpart became defined in the 1980s with the advent of high-resolution computed tomography (HRCT), notably through the work of Staples and Müller [2]. Subsequent updates refined the HRCT criteria for UIP, as reflected in the 2005, 2011, 2018, and 2022 ATS/ERS/JRS/ALAT guidelines [3,4,5,6,7] The typical HRCT features of UIP include subpleural and basal reticulations, traction bronchiectasis, and honeycombing—clusters of cystic airspaces with defined walls, typically 3–10 mm in size, occasionally up to 2.5 cm [8]. In the absence of honeycombing, the pattern is termed “Probable UIP” and may require biopsy or multidisciplinary discussion for definitive diagnosis. While UIP is most often associated with idiopathic pulmonary fibrosis (IPF), it can also occur in secondary interstitial lung diseases (ILDs) such as connective tissue diseases [9,10]. Differentiating primary UIP (pUIP) from secondary UIP (sUIP) is essential, given the divergent therapeutic approaches. Peculiar imaging findings—such as the anterior upper lobe sign [11,12], wedge-shaped fibrosis [13], and exuberant honeycombing [14]—have been linked to autoimmune-related sUIP. IPF management primarily involves antifibrotic agents [15,16], whereas immunosuppressive therapy may be preferred in autoimmune forms [17], with antifibrotics considered in cases of progressive fibrosis [7].

Therefore, the aim of this article is to distinguish radiological differences between primary idiopathic UIP-IPF and sUIP patterns, through expert thoracic radiologist evaluation and advanced AI-driven lung texture analysis in order to improve diagnostic accuracy and treatment.

## 2. Materials and Methods

### 2.1. Population Study

The study included patients assessed at our Regional Centre for Interstitial and Rare Lung Disease; subjects have been retrospectively selected between January 2021 and December 2024, according to all the following inclusion criteria:

• TDiagnosis of Interstitial lung diseases according to the international guidelines published in 2022 [7]; for secondary ILDs related to autoimmune disorders (systemic sclerosis, myositis, Sjögren, etc.), specific rheumatological guidelines were applied.
• At least one HRCT examination—acquired no more than 12 months prior to the Multidisciplinary Discussion; for subjects having multiple HRCT examinations, the latest one was considered for the visual and quantitative assessment.
• Presence of UIP pattern on HRCT examinations.
• Patients were discharged in cases of HRCT examinations with artifacts (motion or respiratory artifacts).

### 2.2. HRCT Protocol

HRCT scans were acquired using the following technical parameters: slice collimation <1.5 mm, gap interval ranging from 0.5 to 1.5 mm, high resolution kernel, 512 × 512 or 768 × 768 resolution matrix. Lung parenchyma was assessed using lung windows (window width 1600 HU, window level −500 HU). Mediastinal strutures (lymphatic nodes, esophageal dilatation, pulmonary-to-aorta ratio, etc.) were evaluated using standard mediastinal window.

### 2.3. Qualitative Evaluation

HRCT examinations were reviewed by 2 radiologists with different level of expertise in thoracic imaging: S.P., a senior thoracic radiologist (with more than >15 years of experience, and M.A., a junior thoracic radiologist in training (with more than >1 year of expertise.

**Figure 1:**
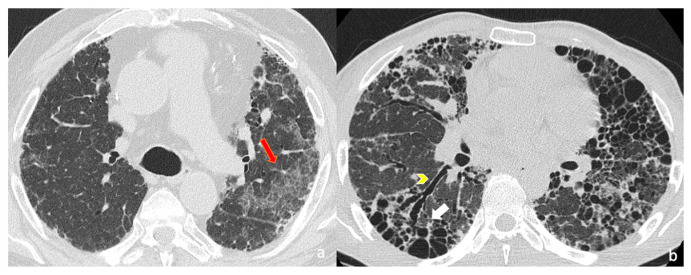
High-resolution computed tomography (HRCT) of the chest in axial (**a**,**b**) views. (**a**) Axial HRCT shows ground-glass opacities (subtle hazy increased attenuation) in left upper lobe, superimposed on fine reticulations (red arrow), suggesting fibrotic interstitial lung disease. (**b**) Advanced fibrotic changes are evident with traction bronchiectasis (yellow arrowhead) and extensive honeycombing (white arrow) in the posterior basal segments of the lower lobes, characterized by clustered cystic airspaces of similar diameter.

Images were evaluated in consensus. The following imaging findings were assessed: (i) fibrosis distribution (evaluating the cranio-caudal extension and the lung involvement in the and axial plane); (ii) key imaging features of fibrosis, represented by honeycomb, traction bronchiectasis and volume loss; (iii) ancillary imaging findings of fibrosis features—reticulations, ground-glass areas and pulmonary ossifications (Figure 1).

**Figure 2:**
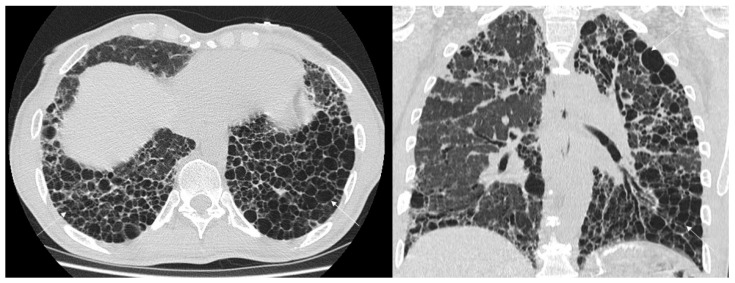
High-resolution computed tomography (HRCT) of the chest in axial (**left**) and coronal (**right**) views showing exuberant honeycombing, characterized by extensive, confluent subpleural cystic airspaces with well-defined walls and similar diameters, predominantly in the lower lobes and posterior regions.

**Figure 3:**
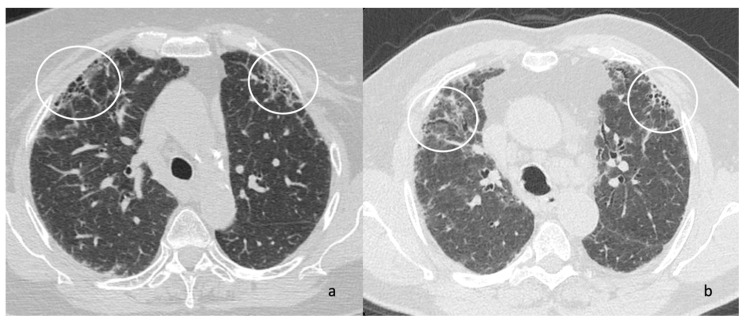
High-resolution computed tomography (HRCT), axial images (**a**,**b**). Predominantly distribution in the ventral-anterior regions of both upper lobes—as clearly demonstrated on Figure 3a,b (white circles).

**Figure 4:**
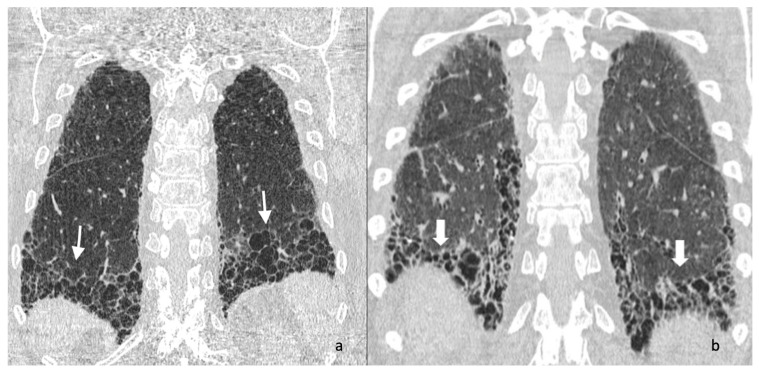
Coronal HRCT images of the chest showing the “straight edge sign” (white arrows in (**a**,**b**), characterized by sharp demarcation of fibrosis with a straight, horizontal lower boundary, limited to the lung bases without significant extension along the lateral margins. This imaging feature is often associated with connective tissue disease-related interstitial lung disease (CTD-ILD) and helps differentiate it from idiopathic pulmonary fibrosis (IPF).

Other more specific fibrosis patterns– including macro/micro honeycombing, exuberant honeycombing (Figure 2), symmetric/asymmetric involvement of abnormalities, the “anterior upper lobe sign” (Figure 3), the “four corner sign”, the “wedge-shaped” distribution (Figure 4), anterior predominance of bronchiectasis (which means i.e., parallel bronchiectasis in the lingular or middle lobe)—were also assessed listed and evaluated. Microcystic and macrocystic honeycombing have been classified according to the definition reported in literature (macro for cystic spaces exceeding 4 mm in diameter) [18]; traction bronchiectasis were scored as follows: mild (=1), moderate (=2) and severe (=3), according to articles published in literature by Walsh et al. [19,20] and Baratella et al. [21]. Lastly, the radiologists analysed the presence of mediastinum abnormalities, such as (i) esophageal dilation; (ii) enlarged size of lymphatic nodes; (iii) pulmonary/aortic ratio. Occasional findings, not necessary directly related to the fibrotic UIP pattern, were also annotated documented by radiologists.

Each qualitative imaging findings was reported by radiologists applying an interpretation consensus; discordant results were discussed together, until the achievement of a full agreement.

### 2.4. Quantitative Evaluation

Quantitative evaluation was performed using a artificial intelligence based lung texture analysis software “Aview Lung Texture” (AVIEW Build 1.1.46.28-win Coreline-©Coreline Soft Co., Ltd. All Rights Reserved).

For quantitative analysis, images with high spatial resolution kernel were processed. The analysis provided—for each patient—total lung volume (expressed in cc), and percentages of different patterns– represented by Honeycomb (H), Reticulations (R), Ground-glass (G), Consolidations (C), Emphysema (E).

### 2.5. Statistical Analysis

Statistical analysis were performed using MedCalc program (MedCalc v. 11.4.4.0, MedCalc Software bvba, Mariakerke, Belgium) and StatPlus program (StatPlus Build 8.0.3/Core v. 7.8.11).

Patient characteristics were reported as: mean (standard deviation) for normally distributed data, median (interquartile range) for non-normally distributed data, or percentages of the relative frequency as appropriate. For main variables, 95% confidence interval (CI) values were reported.

The prevalence of each imaging finding (fibrosis distribution, key imaging of fibrotic pattern), was expressed as percentage.

For qualitative HRCT features, diagnostic accuracy was assessed using a contingency Table 2 × 2. Texture analysis was compared between primary and secondary UIP using a Mann-Whitney U test.

## 3. Results

### 3.1. Qualitative Evaluation

A total of 53 HRCT scans with UIP patterns were retrospectively evaluated in our analysis, including 31 males and 22 females. Demographic and clinical characteristics have been summarized on Table 1.

31 UIPs were radiologically interpreted as “primary UIP”, whereas 22 UIP patterns as “secondary UIP”. The main HRCT features for both groups have been summarized in Table 2. Mean interval time between HRCT examination and diagnosis was equal to 5.61 months (±6).

Fibrosis was predominantly located in the basal regions, with a cranio-caudal distribution observed in many UIP cases (45/53—equal to 84.9%). This typical distribution in 8 cases was not reported (3 pUIPs, 5 sUIPs), whereas it was recognized in almost all pUIP patterns (28/31, equal to 90.3%); in the secondary group, the apico-basal gradient was found in 17 out of 22 cases (77.3%). On axial HRCT images, the peripheral and subpleural distribution was found in 48 cases overall, with only five patients (2 secondary UIPs, 3 primary UIPs) not showing it. Therefore, the percentages observed were similar in both groups (29/31 for pUIPs—equal to 93.5%, and 19/22 sUIPs—86.4%).

The key features of fibrosis were found in almost all UIP patterns; the honeycomb appearance was reported in 45 patients (45/53—84.9%); again, with similar percentages of honeycombing observed in primary and secondary UIP patterns (respectively 27/31—87.1%; 19/22—86.4%).

Subcategorization of honeycombing was additionally performed separating micro-honeycombing and macro-honeycombing in the primary UIP group; among these, macro-honeycombing was found in 15 out of 31 cases whereas micro-honeycombing was observed in 19 cases (33% vs. 40%). In the secondary group the macro-honeycombing was more represented (n = 11) than micro-honeycombing (n = 6), with percentages respectively equal to 50% and 27.3%; in 2 subjects both subtypes of honeycombing was observed.

The presence of exuberant honeycoming was identified in 8 cases of UIPs (15.09%), which were related to IPF (n = 1) and Connective Tissue Diseases (1 SSc, 4 Rheumatoid Arthritis, 1 Sjögren disease, 1 IPAF).

Traction bronchiectasis was found in almost all pUIP patterns: mild 7/31 (22.6%), moderate 18/31 (58%), severe 6/31 (19.4%); in secondary UIP patterns all subjects showed traction bronchiectasis—with severe degree of bronchiectasis observed in 2 out of 22 cases (27.3%). Moderate bronchiectasis and mild bronchiectasis were observed respectively in 12/22 (54.5%) and 8/22 (36.4%) patients whit sUIP patterns.

Reticulations were present in all sUIP cases and in 28 out of 31 pUIPs–100% vs. 84.8%). Among pUIP cases, degree of reticulations was severe in 4 patients (11.8%), moderate in 14 (45.2%) and mild in 13 (41.9%); in the three patients not having reticulations, we have observed mild ground-glass with superimposed tiny reticulations. In the sUIP patterns, they were moderate in 13 (59%) and mild in 9 (41%).

Pulmonary ossifications were reported in 2 pUIP cases.

Wedge shaped fibrosis was found in only 1 secondary UIP case (IV stage of sarcoidosis). The four-corner sign was depicted in only 3 cases: 2 related to a secondary UIP diseases (Sjogren and CTD), and one case related to IPF. Fibrosis was symmetrically distributed in 34 cases (64.2%); an asymmetrical distribution was almost equally distributed in both groups, whit 10 and 9 cases observed in pUIP and sUIP patterns respectively (32,3% vs. 41%). Other parenchymal findings observed in our population included: (i) lobular areas of decreased attenuation, found in four cases (chronic HP, CTD, IPF, familiar fibrosis); (ii) prominent bronchiectasis located at the lingular regions and at the middle lobe were found in 3 cases (2 CTDs, 1 IPF).

Among mediastinal findings, esophageal dilatation was observed in 2 out of 22 secondary cases; similarly, it was reported in 2 IPF cases, due to hiatal hernia.

Pulmonary hypertension (PH)—morphologically defined by a pulmonary artery-to-aorta ratio between main artery and aorta >1- was depicted in 7 out of 53 cases (13.2%); among sUIP group, a value >1 was observed in the following disease: scleroderma (n = 2), rheumatoid arthritis (n = 1), chronic sarcoidosis (n = 1), chronic HP (n = 1). Enlarged lymphatic nodes were noted in 14 out of 31 cases of pUIP (45%); and in 9 cases with sUIP.

### 3.2. Diagnostic Accuracy

Among the 31 patients with a final diagnosis of IPF, 29 were correctly identified by the radiologist as having a primary UIP pattern. In the remaining 2 cases, the final MDD diagnosis was IPF, even if initially the radiological features suggested a secondary UIP pattern. Some of these radiological features—commonly associated to other fibrotic disease (CTDs, fibrotic HP or other non-IPF diseases)—have great specificity but not equal to 100%. Conversely, among the 22 patients with non-IPF ILD, 16 were correctly classified as secondary UIP, while 6 were misclassified as primary UIP. Overall diagnostic accuracy, distinguishing UIP pattern related to IPF from non-IPF diseases, was equal to 84.9%. Sensitivity, specificity, positive predictive value (PPV) and negative predictive value (NPV) were 82.9%, 88.9%, 93.5% and 72.7% respectively.

### 3.3. Quantitative Evaluation

**Figure 5:**
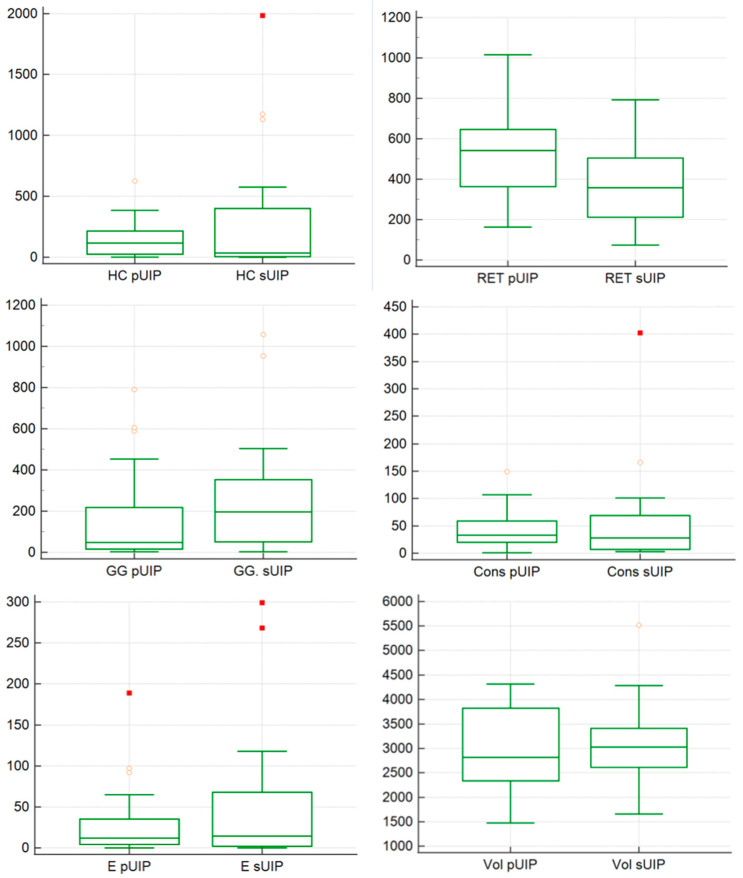
Box whisker plots representing comparisons between primary UIP and secondary UIP—for honeycombing (HC), reticulations (RET), ground-glass (GG), consolidations (Cons), enphysema (E) and Volume (Vol).

Quantitative analysis has been graphically represented on illustrated in Figure 5. The extent of honeycombing, reticulation, ground glass, consolidation and emphysema were compared between the 2 groups—pUIP and sUIP. We did not find. No statistically significant difference in honeycombing extent was observed between the 2 groups with *p* value of 0.85 (mean value of 135.3 in IPF and 314 in non-IPF). Reticulations were significantly more extensive in IPF, with a statistical difference between the 2 groups (*p* = 0.008): a mean value 547.23 (I quartile 376.5; III quartile 643.5) was found among IPF and a value of 371.9 (I quartile 216.5; III quartile 496.25) was reported for non-IPF subjects.

Ground glass distribution reported a statistical difference (*p* = 0.03), with a mean value of 209.04 in IPF (I quartile 17; III 207) and 281.46 in secondary UIP cases (I quartile 71.5; III 350.75).

Consolidations and emphysema were not statistically different between the 2 groups: for consolidation—we found a mean value 56.82 in non-IPF and 40.84 in IPF—with *p* = 0.69; for emphysema, we reported a mean value 52.68 in non-IPF and 28, 30 in IPF *p*—with a value of 0.77. Finally, the amount of normal lung tissue calculated by texture analysis did not differ significantly between groups, with a a mean value 3,095,636 in non-IPF group and a mean value of 2,998,097 in IPF group (*p* = 0.37).

## 4. Discussion

The recognition of an UIP pattern on HRCT imaging is crucial for the patient management and for disease prognosis [22]. According to the Fleischner Society White Paper [5], typical or probable UIP patterns on HRCT are strongly associated with UIP on histology: in the appropriate clinical context, a diagnosis of IPF could be achieved—and no biopsy is required [5]. Therefore, radiologists play a very important role in the diagnosis of interstitial lung diseases: the identification of hallmarks UIP features on HRCT—consisting of honeycombing, reticulations with peripheral bronchiectasis, and basal predominance supports a very high clinical suspicion of IPF.

The identification of an UIP pattern is not only important for the diagnosis, but also for management and prognosis; several radiological studies have clearly demonstrated that patients having a non-IPF disease with UIP pattern on imaging, may exhibit the same prognosis of patients with idiopathic fibrosis [22]. Namely, in the study by Gaxiola et al., patients with Chronic Hypersensitivity Pneumonia (CHP)—actually named Fibrotic Hypersensitivity Pneumonia (fHP)—and radiological and/or histological UIP pattern, exhibited the worst prognosis (with HR value of 4.19, significantly higher than HR observed in NSIP-cases of CHP) [23]. Furthermore, In the study by Nakamura et al., a cohort of subjects with rheumatoid lung diseases has been evaluated analyzing the survival rats associated to the different radiological appearances. Moreover, patients with UIP pattern have shown worse prognosis than those with non-UIP, with a statistical difference reported (*p* = 0.0452) [24].

However, some Authors have demonstrated that UIP patterns found in CTD patients may have better prognosis than UIP-IPF patients [25]. As for these published studies, radiologists should also try to differentiate UIP patterns in idiopathic or secondary causes (connective tissue diseases, fHP). For this reason, our study has investigated the role of radiologists in the differentiation between primary and secondary UIP diseases, emphasizing some radiological features more frequently associated to autoimmune diseases with sUIP pattern—such as the “Anterior Upper Lobe Sign” [11,12], the “Wedged-Shaped Sign” [13], the “Exuberant Honeycombing Sign” [14].

Based on our results, the presence of one or more of the following radiological features—such as anterior upper lobe sign, exuberant honeycombing, wedged-shaped sign, areas of lobular decreased attenuation, pulmonary hypertension, esophageal dilatation—could increase the possibility of an UIP pattern related to a secondary underlying disease. More in detail, the exuberant macro-honeycombing was detected in 7 UIP related to secondary diseases (7/22, equal to a percentage of 31.8%)—reinforcing its association with rheumatoid arthritis. In addition to exuberant honeycombing, other specific radiological signs of fibrosis provided critical insights. The “Four Corner Sign” was identified in two patients with UIP: one associated with Sjogren’s syndrome and the other related to mixed connective tissue diseases. Pulmonary hypertension has been detected among UIP related to a secondary disease in 5 cases (22.7%): in our analysis, it was the most frequent radiological after the exuberant HC.

Indeed, based on our results, the presence of exuberant honeycombing and/or decreased lobular attenuation, and/or pulmonary hypertension, and/or esophageal dilatation, was useful to correctly classify 11 out of 22 cases (50%) having a non-IPF UIP.

The results of our study underscore the indispensable role of the expert thoracic radiologist in distinguishing between IPF related to IPF and UIP related to secondary diseases. Interestingly, in all cases the quantitative analysis evaluates the typical hallmarks of fibrotic UIP patterns, mainly consisting of honeycombing and reticulations. However, the quantitative AI evaluation demonstrates limitations in its ability to provide characterization of certain radiological fibrotic features: the exuberant and macrocystic honeycombing were not specifically labeled or distinguished in our quantitative analysis. AI’s algorithms—which excel in detecting standard fibrotic patterns such as honeycombing and reticulations—struggled to differentiate macro-honeycombing from micro-honeycombing as they are not able to give these qualitative/subcategorization results. This limitation is of clinical importance since exuberant form of honeycomb could be a key indicator of secondary UIP. The inability of AI to distinguish between different types of honeycombing likely stems from its reliance on pixel-based pattern recognition without a nuanced understanding of the morphological characteristics that define macro-honeycombing, such as the size, distribution, and extent of cystic airspaces. Considering the four corner sign, this particular fibrotic distribution is still not categorized by many AI tools. Expert thoracic radiologists, in contrast, could accurately identify these features, highlighting the critical role of human expertise in refining the differential diagnosis. Considering quantitative results, reticulations were more prevalent in UIP patterns associated with IPF; ground-glass areas were conversely more found in secondary UIP patterns. These results do not have high importance, since our study has enrolled a limited number of patients: a possible speculation, to explain more ground-glass areas in secondary UIP patterns, could be the presence of a certain degree of inflammation in the lungs. Reticulations are more represented in primary UIP patterns, and this could be related to the presence of UIP patterns different from those observed in secondary diseases. In the latter, UIP patterns are more reproduced by cystic changes, and less reticulations may be found.

However, the importance of AI in the assessment of ILD has been recently demonstrated. AI models allow clinicians and radiologists in the classification of ILD subtypes, which may be challenging, especially for those with low level of expertise in ILD management [26]. AI-based methods will increasingly complement expert visual assessment and multidisciplinary discussion (MDD), improving consistency and objectivity—particularly in centers lacking specialized thoracic radiologists [27].

Our study has different limitations. First of all, it includes a small number of cases, so that results obtained may have limited values. Secondarily, secondary UIP include a large spectrum of diseases: some radiological features, in example, are typically more depicted in CTD cases, and not in fHP. Therefore, the differentiation between UIP patterns should be evaluated in large series, and comparing restricted groups of secondary diseases. Lastly, our retrospective evaluation didn’t include biopsy or data from bronchoalveolar lavage fluid since we have predominantly collected cases with HRCT diagnosis of UIP: our evaluation lack of cases with atypical radiological presentation, and histological diagnosis of UIP.

## 5. Conclusions

The expert thoracic radiologist still plays a relevant role in identifying whether a UIP pattern refers to a pUIP or sUIP, by meticulously evaluating hallmark imaging features. While AI-driven lung texture analysis offers valuable quantitative data, it has limits in distinguishing between macro- and micro-honeycomb or in consistently recognizing patterns like the four-corner sign. For this reason, targeted training provided by expert thoracic radiologist might implement diagnostic capabilities of AI-driven lung texture software in detect key symbols, besides the quantitative analysis already provided.

In conclusion, synergetic approach between human expertise and AI leads to an optimal diagnostic workflow able to enhance prompt therapy and patient outcomes.

## Figures and Tables

**Table 1:** Main demographic and clinical characteristics of population study.

	pUIPs (n = 31)	sUIPs (n = 22)
Age	73.3 years (range 51–88)	74.3 years (range 40–86)
Gender	64.5% (20/31) males; 35.5% (11/31) females	50% (11/22) males; 50% (11/22) females
Antibody Positivity	19.4% (6/31)	72.3% (16/22)
Non Smokers	38.7% (12/31)	36.3% (8/22)
Current or Former Smokers	61.2% (19/31)	63.6% (14/22)
Pack-Years (Mean)	15.1	14.6

**Table 2:** HRCT findings in IPF subjects (p-UIP) and patients with UIP related to other secondary interstitial diseases (s-UIP).

HRCT Findings	p-UIP	s-UIP
Cranio-caudal fibrosis distribution	28/31 (90.3%)	17/22 (77.3%)
Subpleural/peripheral distribution	29/31 (93.5%)	19/22 (86.4%)
Honeycombing - macro-HC - micro-HC	27/31 (87.1%)	19/22 (86.4%)
	
	15/31 (33%)	11/22 (50%)
	19/31 (40%)	6/22 (27.3%)
Exhuberant Honeycoming	1/31 (3.2%)	7/22 (31.8%)
Traction bronchiectasis	31/31 (100%)	22/22 (100%)
mild	7/31 (22.6%)	8/22 (36.4%)
moderate	18/31 (58%)	12/22 (54.5%
severe	6/31 (19.4%)	2/22 (9.09%)
Reticulations	28/31 (84.8%)	22/22 (100%)
mild	13/31 (41.9%)	9/22 (40.9%)
moderate	14/31 (45.2%)	13/22 (59.1%)
severe	4/31 (11.8%)	0/22 (0%)
Pulmonary ossifications	2/31 (6.5%)	0/22
Wedge-shaped fibrosis	0/31	1/22 (4.5%)
Four-corner sign	1/31 (3.2%)	2/22 (9%)
Oesophagus dilatation	2/31 (6.5%) *	2/31 (6.5%)
Pulmonary hypertension	2/31 (6.5%)	5/22 (22.7%)

* related to esophageal-gastric junction hernia.

## Data Availability

The data presented in this study are available upon request from the corresponding author. The data are not publicly available due to ethical reasons.

## Abbreviations

The following abbreviations are used in this manuscript:

UIP	Usual Interstitial Pneumonia
HRCT	High Resolution Computed Tomography
ILD	Interstitial Lung Disease
GGO	Ground Glass Opacity
CT	Computed Tomography
IPF	Idiopathic Pulmonary Fibrosis
HU	Hounsfield Unit
CI	Confidence Interval
CTD	Connective Tissue Disease
IPAF	Interstitial Pneumonia with Autoimmune Feature
NSIP	Non Specific Interstitial Pneumonia
CHP	Chronic Hypersensitivity Pneumonia
fHP	Fibrotic Hypersensitivity Pneumonia
AI	Artificial Intelligence
